# *N*′-(2-Bromo-5-hy­droxy-4-meth­oxy­benzyl­idene)-3,5-dihy­droxy­benzo­hydrazide methanol monosolvate

**DOI:** 10.1107/S1600536811008695

**Published:** 2011-03-12

**Authors:** Zong-Gui Wang, Ling Yuan, Li Zhou, Yi Nan

**Affiliations:** a The Second Hospital of Jilin University, Jilin Province 130041, People’s Republic of China; b Pharmacy College of Ningxia Medical University, Ningxia Province 750004, People’s Republic of China; c Minority Traditional Medical Center of Minzu University of China, Beijing 100081, People’s Republic of China; d Traditional Chinese Medicine College of Ningxia Medical University, Ningxia Province 750004, People’s Republic of China

## Abstract

In the crystal structure of the title compound, C_15_H_13_BrN_2_O_5_·CH_3_OH, the methanol solvent mol­ecule links symmetry-related mol­ecules through O—H⋯O and N—H⋯O hydrogen bonds. Further inter­molecular O—H⋯O hydrogen bonds link symmetry-related mol­ecules, leading to the formation of a three-dimensional network. Two of the H atoms involved in hydrogen bonding are disordered. The dihedral angle between the rings is 5.64 (14)°.

## Related literature

The title compound is a Schiff base with potential anti­bacterial properties. For the anti­bacterial and anti­tumor activity of Schiff base complexes, see: Brückner *et al.* (2000 ▶); Harrop *et al.* (2003 ▶); Ren *et al.* (2002 ▶). For related structures, see: Diao (2007 ▶); Diao *et al.* (2007 ▶); Huang *et al.* (2007 ▶); Li *et al.* (2007 ▶).
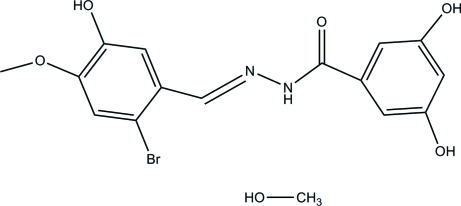


## Experimental

### 

#### Crystal data

C_15_H_13_BrN_2_O_5_·CH_4_O
*M* *_r_* = 413.23
Monoclinic, 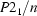

*a* = 7.4242 (17) Å
*b* = 17.709 (4) Å
*c* = 12.927 (3) Å
β = 96.493 (3)°
*V* = 1688.7 (6) Å^3^
*Z* = 4
Mo *K*α radiation
μ = 2.47 mm^−1^
*T* = 296 K
0.47 × 0.24 × 0.19 mm

#### Data collection

Bruker SMART 1000 CCD diffractometer
Absorption correction: multi-scan (*SADABS*; Sheldrick, 2003 ▶) *T*_min_ = 0.488, *T*_max_ = 0.616
9884 measured reflections
3780 independent reflections
2569 reflections with *I* > 2σ(*I*)
*R*_int_ = 0.035

#### Refinement

*R*[*F* ^2^ > 2σ(*F* ^2^)] = 0.043
*wR*(*F* ^2^) = 0.111
*S* = 1.01
3780 reflections
231 parameters
H-atom parameters constrained
Δρ_max_ = 0.46 e Å^−3^
Δρ_min_ = −0.36 e Å^−3^

### 

Data collection: *SMART* (Bruker, 2001 ▶); cell refinement: *SAINT-Plus* (Bruker, 2003 ▶); data reduction: *SAINT-Plus*; program(s) used to solve structure: *SHELXTL* (Sheldrick, 2008 ▶); program(s) used to refine structure: *SHELXTL*; molecular graphics: *SHELXTL*; software used to prepare material for publication: *SHELXTL*.

## Supplementary Material

Crystal structure: contains datablocks I, global. DOI: 10.1107/S1600536811008695/kj2170sup1.cif

Structure factors: contains datablocks I. DOI: 10.1107/S1600536811008695/kj2170Isup2.hkl

Additional supplementary materials:  crystallographic information; 3D view; checkCIF report

## Figures and Tables

**Table 1:** Hydrogen-bond geometry (Å, °)

*D*—H⋯*A*	*D*—H	H⋯*A*	*D*⋯*A*	*D*—H⋯*A*
O2—H2*A*⋯O5^i^	0.82	2.11	2.932 (3)	180
O2—H2*B*⋯O1^ii^	0.82	2.33	3.148 (4)	180
O4—H4*A*⋯O3^iii^	0.82	1.84	2.655 (3)	175
O5—H5*A*⋯O5^iv^	0.82	2.15	2.859 (5)	145
O5—H5*B*⋯O2^v^	0.82	2.12	2.932 (3)	171
N2—H2⋯O6	0.86	2.15	2.984 (4)	164
O6—H6⋯O4^vi^	0.82	2.33	3.101 (4)	157

Symmetry codes: (i) 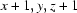; (ii) 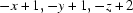; (iii) 
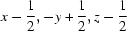
; (iv) 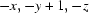; (v) 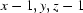; (vi) 
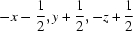
.

**Fig. 1.**
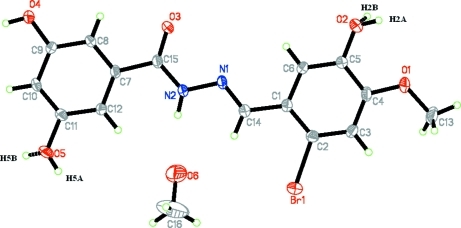
The molecular structure of the title compound. Thermal ellipsoids are shown at 30% probability level.

**Fig. 2.**
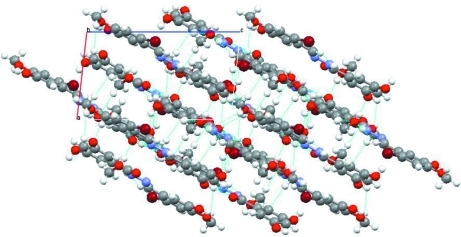
Crystal packing of the title compound, viewed down the b axis. The dashed lines represent hydrogen bonding interactions.

## supplementary crystallographic information

### Comment

Schiff base compounds have received much attention in recent years. Some of the complexes have been found to have antibacterial and antitumor properties (Brückner,*et al.*,2000; Harrop *et al.*,2003; Ren *et al.*,2002). As part of our research programme on Schiff base compounds (Diao, 2007; Diao *et al.*, 2007; Li *et al.*, 2007; Huang *et al.*, 2007), we report here the structure of the title compound.

The title compound co-crystallizes with one methanol molecule (Fig. 1), which links symmetry-related molecules through O—H···O and one N—H···O hydrogen bonds. In the crystal structure, further intermolecular O—H···O hydrogen bonds link symmetry-related molecules (Table 1), forming a three-dimensional network (Fig.2). The H atoms bonded to O2 and O5 are disordered over two positions. All positions take part in intermolecular hydrogen bonds.

### Experimental

2-Bromo-5-hydroxy-4-methoxybenzaldehyde (0.1 mmol, 23.1 mg) and 3,5-Dihydroxybenzhydrazide (0.1 mmol, 16.8 mg) were dissolved in a methanol solution (10 ml). The mixture was stirred at room temperature for 1 h and filtered. After keeping the filtrate in air for three days, colorless block-like crystals were formed.

### Refinement

The H2 atom bonded to N2 was located in a difference map and refined freely, other H atoms were placed in geometrically idealized positions and allowed to ride on their parent atoms, O—H=0.82,*C*—H=0.93 for phenyl, 0.96 for methyl H atoms, with *U*_iso_(H)=1.2*U*_eq_(C) for phenyl and 1.5eqU(C) for methyl and hydroxyl groups. H atoms bonded to O2 and O5 were split over two positions with a fixed occupation factor of 0.5.

### Crystal data

**Table:**

C_15_H_13_BrN_2_O_5_·CH_4_O	*F*(000) = 840
*M**_r_* = 413.23	*D*_x_ = 1.625 Mg m^−^^3^
Monoclinic, *P*2_1_/*n*	Mo *K*α radiation, λ = 0.71073 Å
Hall symbol: -P 2yn	Cell parameters from 2413 reflections
*a* = 7.4242 (17) Å	θ = 2.3–25.8°
*b* = 17.709 (4) Å	µ = 2.47 mm^−^^1^
*c* = 12.927 (3) Å	*T* = 296 K
β = 96.493 (3)°	Block, colorless
*V* = 1688.7 (6) Å^3^	0.47 × 0.24 × 0.19 mm
*Z* = 4	

### Data collection

**Table:**

Bruker SMART 1000 CCD diffractometer	3780 independent reflections
Radiation source: fine-focus sealed tube	2569 reflections with *I* > 2σ(*I*)
graphite	*R*_int_ = 0.035
φ and ω scans	θ_max_ = 27.2°, θ_min_ = 2.0°
Absorption correction: multi-scan (*SADABS*; Sheldrick, 2003)	*h* = −9→9
*T*_min_ = 0.488, *T*_max_ = 0.616	*k* = −14→22
9884 measured reflections	*l* = −16→16

### Refinement

**Table:**

Refinement on *F*^2^	Primary atom site location: structure-invariant direct methods
Least-squares matrix: full	Secondary atom site location: difference Fourier map
*R*[*F*^2^ > 2σ(*F*^2^)] = 0.043	Hydrogen site location: inferred from neighbouring sites
*wR*(*F*^2^) = 0.111	H-atom parameters constrained
*S* = 1.01	*w* = 1/[σ^2^(*F*_o_^2^) + (0.0482*P*)^2^ + 0.8829*P*] where *P* = (*F*_o_^2^ + 2*F*_c_^2^)/3
3780 reflections	(Δ/σ)_max_ = 0.001
231 parameters	Δρ_max_ = 0.46 e Å^−^^3^
0 restraints	Δρ_min_ = −0.36 e Å^−^^3^

### Special details

**Table:**

Geometry. All e.s.d.'s (except the e.s.d. in the dihedral angle between two l.s. planes) are estimated using the full covariance matrix. The cell e.s.d.'s are taken into account individually in the estimation of e.s.d.'s in distances, angles and torsion angles; correlations between e.s.d.'s in cell parameters are only used when they are defined by crystal symmetry. An approximate (isotropic) treatment of cell e.s.d.'s is used for estimating e.s.d.'s involving l.s. planes.
Refinement. Refinement of *F*^2^ against ALL reflections. The weighted *R*-factor *wR* and goodness of fit *S* are based on *F*^2^, conventional *R*-factors *R* are based on *F*, with *F* set to zero for negative *F*^2^. The threshold expression of *F*^2^ > σ(*F*^2^) is used only for calculating *R*-factors(gt) *etc*. and is not relevant to the choice of reflections for refinement. *R*-factors based on *F*^2^ are statistically about twice as large as those based on *F*, and *R*- factors based on ALL data will be even larger.

### Fractional atomic coordinates and isotropic or equivalent isotropic displacement parameters (Å^2^)

**Table:**

	*x*	*y*	*z*	*U*_iso_*/*U*_eq_	Occ. (<1)
Br1	0.38821 (6)	0.687078 (19)	0.54748 (3)	0.05529 (16)	
O1	0.6149 (3)	0.58020 (13)	0.91725 (17)	0.0527 (6)	
O2	0.5254 (4)	0.43891 (13)	0.86339 (18)	0.0652 (8)	
H2A	0.6165	0.4422	0.9054	0.098*	0.50
H2B	0.4884	0.4338	0.9204	0.098*	0.50
O3	0.1127 (4)	0.31210 (12)	0.42579 (17)	0.0541 (7)	
O4	−0.2407 (4)	0.19793 (12)	0.12235 (16)	0.0501 (6)	
H4A	−0.2804	0.1933	0.0609	0.075*	
O5	−0.1488 (4)	0.45108 (14)	0.01335 (18)	0.0636 (8)	
H5A	−0.0942	0.4914	0.0180	0.095*	0.50
H5B	−0.2327	0.4458	−0.0330	0.095*	0.50
N1	0.2142 (3)	0.44876 (14)	0.49680 (17)	0.0348 (6)	
N2	0.1270 (3)	0.43712 (14)	0.39748 (18)	0.0354 (6)	
H2	0.1071	0.4774	0.3608	0.059 (11)*	
C1	0.3706 (4)	0.53397 (16)	0.6178 (2)	0.0314 (7)	
C2	0.4255 (4)	0.60688 (16)	0.6447 (2)	0.0327 (7)	
C3	0.5083 (4)	0.62529 (17)	0.7431 (2)	0.0370 (7)	
H3A	0.5428	0.6748	0.7590	0.044*	
C4	0.5387 (4)	0.56937 (17)	0.8169 (2)	0.0373 (7)	
C5	0.4914 (5)	0.49492 (17)	0.7916 (2)	0.0394 (8)	
C6	0.4093 (4)	0.47768 (17)	0.6937 (2)	0.0367 (7)	
H6A	0.3787	0.4278	0.6774	0.044*	
C7	−0.0144 (4)	0.35570 (16)	0.2601 (2)	0.0311 (7)	
C8	−0.0809 (4)	0.28403 (17)	0.2367 (2)	0.0343 (7)	
H8A	−0.0643	0.2459	0.2863	0.041*	
C9	−0.1723 (4)	0.26884 (16)	0.1392 (2)	0.0323 (7)	
C10	−0.1920 (4)	0.32480 (16)	0.0639 (2)	0.0326 (7)	
H10A	−0.2497	0.3146	−0.0023	0.039*	
C11	−0.1249 (4)	0.39546 (17)	0.0888 (2)	0.0369 (7)	
C12	−0.0367 (4)	0.41262 (16)	0.1864 (2)	0.0345 (7)	
H12A	0.0062	0.4611	0.2019	0.041*	
C13	0.6728 (5)	0.6544 (2)	0.9486 (3)	0.0553 (10)	
H13A	0.7628	0.6715	0.9062	0.083*	
H13B	0.7237	0.6536	1.0203	0.083*	
H13C	0.5710	0.6882	0.9406	0.083*	
C14	0.2758 (4)	0.51463 (17)	0.5154 (2)	0.0350 (7)	
H14A	0.2607	0.5512	0.4636	0.042*	
C15	0.0798 (4)	0.36657 (17)	0.3681 (2)	0.0329 (7)	
O6	0.0298 (5)	0.58875 (17)	0.3072 (3)	0.0832 (9)	
H6	−0.0533	0.6068	0.3359	0.125*	
C16	0.0995 (12)	0.6427 (4)	0.2480 (5)	0.156 (4)	
H16A	0.0079	0.6588	0.1942	0.234*	
H16B	0.2005	0.6222	0.2171	0.234*	
H16C	0.1391	0.6850	0.2911	0.234*	

### Atomic displacement parameters (Å^2^)

**Table:**

	*U*^11^	*U*^22^	*U*^33^	*U*^12^	*U*^13^	*U*^23^
Br1	0.0774 (3)	0.0372 (2)	0.0484 (2)	−0.00523 (18)	−0.00500 (17)	0.01134 (15)
O1	0.0713 (17)	0.0449 (14)	0.0359 (12)	−0.0053 (12)	−0.0200 (11)	−0.0098 (10)
O2	0.103 (2)	0.0426 (14)	0.0412 (13)	−0.0058 (14)	−0.0278 (13)	0.0078 (11)
O3	0.0851 (19)	0.0325 (12)	0.0368 (12)	−0.0003 (12)	−0.0268 (12)	0.0045 (10)
O4	0.0834 (19)	0.0339 (13)	0.0286 (11)	−0.0130 (11)	−0.0126 (12)	−0.0003 (9)
O5	0.094 (2)	0.0457 (15)	0.0418 (13)	−0.0179 (13)	−0.0316 (13)	0.0206 (11)
N1	0.0415 (15)	0.0358 (14)	0.0241 (12)	0.0030 (12)	−0.0085 (11)	−0.0049 (10)
N2	0.0475 (16)	0.0300 (13)	0.0255 (12)	0.0015 (11)	−0.0101 (11)	−0.0012 (11)
C1	0.0342 (17)	0.0315 (16)	0.0276 (14)	−0.0011 (12)	0.0001 (12)	−0.0036 (12)
C2	0.0336 (17)	0.0299 (15)	0.0343 (15)	0.0019 (13)	0.0024 (13)	0.0029 (12)
C3	0.0408 (19)	0.0288 (16)	0.0398 (17)	−0.0038 (13)	−0.0030 (14)	−0.0074 (13)
C4	0.0387 (19)	0.0387 (17)	0.0320 (16)	0.0009 (14)	−0.0075 (13)	−0.0084 (13)
C5	0.052 (2)	0.0307 (16)	0.0328 (16)	0.0003 (14)	−0.0086 (14)	−0.0008 (13)
C6	0.0444 (19)	0.0268 (15)	0.0365 (16)	−0.0030 (13)	−0.0058 (14)	−0.0042 (12)
C7	0.0333 (17)	0.0329 (16)	0.0250 (14)	0.0037 (13)	−0.0058 (12)	−0.0037 (12)
C8	0.0463 (19)	0.0302 (15)	0.0244 (14)	0.0036 (13)	−0.0050 (13)	0.0011 (12)
C9	0.0408 (19)	0.0263 (15)	0.0286 (15)	0.0003 (13)	−0.0015 (13)	−0.0041 (12)
C10	0.0369 (17)	0.0381 (17)	0.0205 (13)	−0.0013 (13)	−0.0065 (12)	0.0003 (12)
C11	0.0421 (19)	0.0345 (17)	0.0315 (15)	−0.0014 (14)	−0.0074 (13)	0.0074 (13)
C12	0.0417 (18)	0.0279 (15)	0.0306 (15)	−0.0030 (13)	−0.0101 (13)	−0.0007 (12)
C13	0.058 (2)	0.050 (2)	0.054 (2)	−0.0051 (18)	−0.0111 (18)	−0.0216 (18)
C14	0.0421 (19)	0.0341 (16)	0.0273 (15)	−0.0006 (14)	−0.0020 (13)	−0.0013 (12)
C15	0.0357 (18)	0.0360 (16)	0.0251 (14)	0.0019 (13)	−0.0052 (12)	−0.0031 (12)
O6	0.093 (3)	0.0608 (19)	0.095 (2)	0.0126 (16)	0.0061 (19)	0.0034 (17)
C16	0.276 (10)	0.091 (5)	0.119 (5)	0.061 (5)	0.094 (6)	0.051 (4)

### Geometric parameters (Å, °)

**Table:**

Br1—C2	1.896 (3)	C4—C5	1.394 (4)
O1—C4	1.368 (3)	C5—C6	1.375 (4)
O1—C13	1.428 (4)	C6—H6A	0.9300
O2—C5	1.362 (4)	C7—C8	1.383 (4)
O2—H2A	0.8200	C7—C12	1.384 (4)
O2—H2B	0.8200	C7—C15	1.501 (4)
O3—C15	1.227 (3)	C8—C9	1.388 (4)
O4—C9	1.363 (3)	C8—H8A	0.9300
O4—H4A	0.8200	C9—C10	1.385 (4)
O5—C11	1.383 (3)	C10—C11	1.372 (4)
O5—H5A	0.8200	C10—H10A	0.9300
O5—H5B	0.8200	C11—C12	1.388 (4)
N1—C14	1.266 (4)	C12—H12A	0.9300
N1—N2	1.386 (3)	C13—H13A	0.9600
N2—C15	1.341 (4)	C13—H13B	0.9600
N2—H2	0.8600	C13—H13C	0.9600
C1—C2	1.386 (4)	C14—H14A	0.9300
C1—C6	1.405 (4)	O6—C16	1.362 (6)
C1—C14	1.468 (4)	O6—H6	0.8200
C2—C3	1.388 (4)	C16—H16A	0.9600
C3—C4	1.376 (4)	C16—H16B	0.9600
C3—H3A	0.9300	C16—H16C	0.9600
			
C4—O1—C13	118.5 (3)	C7—C8—H8A	119.9
C5—O2—H2A	118.4	C9—C8—H8A	119.9
C5—O2—H2B	129.6	O4—C9—C10	122.6 (3)
H2A—O2—H2B	75.4	O4—C9—C8	117.3 (2)
C9—O4—H4A	109.5	C10—C9—C8	120.1 (3)
C11—O5—H5A	123.0	C11—C10—C9	118.8 (3)
C11—O5—H5B	117.4	C11—C10—H10A	120.6
H5A—O5—H5B	118.8	C9—C10—H10A	120.6
C14—N1—N2	115.7 (2)	C10—C11—O5	118.0 (3)
C15—N2—N1	118.7 (2)	C10—C11—C12	122.3 (3)
C15—N2—H2	126.4	O5—C11—C12	119.7 (3)
N1—N2—H2	114.8	C7—C12—C11	118.3 (3)
C2—C1—C6	117.1 (3)	C7—C12—H12A	120.9
C2—C1—C14	122.7 (3)	C11—C12—H12A	120.9
C6—C1—C14	120.2 (3)	O1—C13—H13A	109.5
C1—C2—C3	122.4 (3)	O1—C13—H13B	109.5
C1—C2—Br1	121.0 (2)	H13A—C13—H13B	109.5
C3—C2—Br1	116.6 (2)	O1—C13—H13C	109.5
C4—C3—C2	119.2 (3)	H13A—C13—H13C	109.5
C4—C3—H3A	120.4	H13B—C13—H13C	109.5
C2—C3—H3A	120.4	N1—C14—C1	120.8 (3)
O1—C4—C3	125.0 (3)	N1—C14—H14A	119.6
O1—C4—C5	114.9 (3)	C1—C14—H14A	119.6
C3—C4—C5	120.0 (3)	O3—C15—N2	122.2 (3)
O2—C5—C6	119.6 (3)	O3—C15—C7	120.3 (3)
O2—C5—C4	120.4 (3)	N2—C15—C7	117.5 (2)
C6—C5—C4	120.0 (3)	C16—O6—H6	109.5
C5—C6—C1	121.3 (3)	O6—C16—H16A	109.5
C5—C6—H6A	119.4	O6—C16—H16B	109.5
C1—C6—H6A	119.4	H16A—C16—H16B	109.5
C8—C7—C12	120.4 (2)	O6—C16—H16C	109.5
C8—C7—C15	116.0 (2)	H16A—C16—H16C	109.5
C12—C7—C15	123.6 (3)	H16B—C16—H16C	109.5
C7—C8—C9	120.2 (3)		
			
C14—N1—N2—C15	171.4 (3)	C15—C7—C8—C9	179.1 (3)
C6—C1—C2—C3	−2.7 (5)	C7—C8—C9—O4	−177.4 (3)
C14—C1—C2—C3	177.2 (3)	C7—C8—C9—C10	2.1 (5)
C6—C1—C2—Br1	177.9 (2)	O4—C9—C10—C11	177.5 (3)
C14—C1—C2—Br1	−2.2 (4)	C8—C9—C10—C11	−2.0 (5)
C1—C2—C3—C4	0.5 (5)	C9—C10—C11—O5	−178.8 (3)
Br1—C2—C3—C4	179.9 (2)	C9—C10—C11—C12	0.5 (5)
C13—O1—C4—C3	−1.8 (5)	C8—C7—C12—C11	−0.8 (5)
C13—O1—C4—C5	177.9 (3)	C15—C7—C12—C11	179.4 (3)
C2—C3—C4—O1	−178.4 (3)	C10—C11—C12—C7	0.9 (5)
C2—C3—C4—C5	1.9 (5)	O5—C11—C12—C7	−179.8 (3)
O1—C4—C5—O2	−1.5 (5)	N2—N1—C14—C1	179.9 (3)
C3—C4—C5—O2	178.3 (3)	C2—C1—C14—N1	−173.9 (3)
O1—C4—C5—C6	178.3 (3)	C6—C1—C14—N1	5.9 (5)
C3—C4—C5—C6	−2.0 (5)	N1—N2—C15—O3	−0.7 (5)
O2—C5—C6—C1	179.5 (3)	N1—N2—C15—C7	−180.0 (3)
C4—C5—C6—C1	−0.3 (5)	C8—C7—C15—O3	8.3 (4)
C2—C1—C6—C5	2.5 (5)	C12—C7—C15—O3	−171.9 (3)
C14—C1—C6—C5	−177.3 (3)	C8—C7—C15—N2	−172.4 (3)
C12—C7—C8—C9	−0.7 (5)	C12—C7—C15—N2	7.3 (5)

### Hydrogen-bond geometry (Å, °)

**Table:**

*D*—H···*A*	*D*—H	H···*A*	*D*···*A*	*D*—H···*A*
O2—H2A···O5^i^	0.82	2.11	2.932 (3)	180
O2—H2B···O1^ii^	0.82	2.33	3.148 (4)	180
O4—H4A···O3^iii^	0.82	1.84	2.655 (3)	175
O5—H5A···O5^iv^	0.82	2.15	2.859 (5)	145
O5—H5B···O2^v^	0.82	2.12	2.932 (3)	171
N2—H2···O6	0.86	2.15	2.984 (4)	164
O6—H6···O4^vi^	0.82	2.33	3.101 (4)	157

Symmetry codes: (i) *x*+1, *y*, *z*+1; (ii) −*x*+1, −*y*+1, −*z*+2; (iii) *x*−1/2, −*y*+1/2, *z*−1/2; (iv) −*x*, −*y*+1, −*z*; (v) *x*−1, *y*, *z*−1; (vi) −*x*−1/2, *y*+1/2, −*z*+1/2.
